# iDEP-based single-cell isolation in a two-dimensional array of chambers addressed by easy-to-align wireless electrodes†

**DOI:** 10.1039/d4lc00976b

**Published:** 2025-02-14

**Authors:** Thilini N. Rathnaweera, Robbyn K. Anand

**Affiliations:** a Department of Chemistry, Iowa State University Ames Iowa 50011 USA rkanand@iastate.edu

## Abstract

Platforms capable of selective single-cell capture and enclosure in a fluidically isolated volume for subsequent analysis are crucial for unmasking cellular heterogeneity. Our research group has previously reported an approach that employs wireless bipolar electrodes (BPEs) to facilitate individual isolation of cells in large arrays of pico- to nanoliter scale chambers by dielectrophoresis (DEP). This device was leveraged for a single-cell enzymatic assay and the isolation of circulating tumor cells (CTCs) from patient-derived blood samples, which takes advantage of the selectivity of DEP. However, alignment of BPEs to the microchamber openings is nontrivial, and augmentation of the array dimensions accumulates alignment error, thereby disrupting the uniformity of cell capture across the device. Thus, tolerance-forgiving designs that are simultaneously expandable are in demand. To address this demand, we present an approach that combines BPEs with insulator DEP (iDEP) to drastically expand alignment tolerance. This iDEP-BPE device offers a vertical tolerance (the distance the BPE is recessed within each microchamber) of 80 μm while the horizontal tolerance is nearly infinite. Further, the iDEP-BPE device decreases the exposure of cells to electrode surfaces and reactive oxygen species, thereby preserving their viability. Finally, this iDEP approach can be carried out with BPEs that are easy to fabricate, lacking features that require high-resolution lithography. These advancements potentiate the broad adoption of the iDEP-BPE approach for selective single-cell capture and on-chip analysis and potentiate its commercialization upon deployment of appropriate thermoplastic materials.

## Abstract 2

An easily alignable platform for selective single-cell capture and confinement using insulator-based dielectrophoresis.

## Graphical abstract

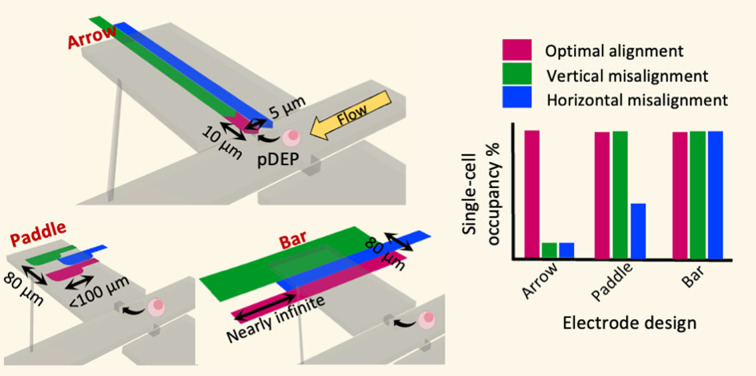

## Introduction

Cellular heterogeneity refers to the variations that exist among cells within the same population. In the past, research and clinical accomplishments were facilitated by population-averaged cellular information. However, the recognition of cell-to-cell variability has transformed our understanding of disease complexity, revealing previously unappreciated heterogeneity and opening new avenues for research and therapeutic development. For instance, in the context of cancer, the choice of a therapeutic approach must consider cellular heterogeneity, and the efficacy of a treatment frequently hinges on the response exhibited by a specific subset of tumor cells. Cellular heterogeneity encompasses both genetic and phenotypic variations, among which are cellular secretion,^1^ expression of surface proteins,^2^ mRNA expression^3^ and deformability.^4^ However, it is worth noting that the efficiency of cell isolation and retention along with the capability of complete segregation allows reliable single-cell measurements. To this end, we have previously demonstrated that cells can be individually isolated and sealed-off in chambers on a valve-free microfluidic device.^5,6^ Further, our integrated approach offers the opportunity to perform on-chip analysis (*i.e.*, live-cell assays or lysed-cell assays) immediately following cell isolation thereby avoiding cell-transfer steps and the need for multiple platforms.

Among a plethora of single-cell isolation techniques, immunoaffinity-based methods^7,8^ are widely employed but have limitations. For instance, in the isolation of circulating tumor cells (CTCs) from blood, epithelial cell adhesion molecules (EpCAM) are commonly targeted.^9,10^ However, EpCAM is found to be downregulated during epithelial mesenchymal transition (EMT) thereby making such single marker-based isolation over selective. Further, such labelling techniques are laborious, time and reagent consuming, and can be only applied when unique surface markers are available.^11^ Alternatively, passive isolation techniques such as filtration^12,13^ and deterministic lateral displacement (DLD)^14^ have also been reported. Nevertheless, the techniques that exclusively harness cell size for isolation are under selective. Importantly, there is much overlap identified between CTCs and background leukocytes thus yielding impure samples for targeted analysis.^15^ Therefore, it is essential for the cell isolation method to be appropriately selective to prevent bias.

To this end, label-free techniques such as dielectrophoresis (DEP) become beneficial. Dielectrophoretic cell separation is driven by unique dielectric signatures of cells that are known to arise from distinct factors including glycosylation^16^ and the extent of membrane folding.^17^ Thus, DEP offers superior selectivity in cell separation and has been exploited in various single-cell studies. The Jiang group has utilized nDEP forces to separate flowing cells from particles and to trap single cells on an array of bipolar electrodes (BPEs).^18^ While this integrated device offers a single-cell occupancy rate of 72%, it is worth noting that the carefully selected cells are released upon disconnecting the voltage supply, due to the lack of physical barriers for confinement. In contrast, the Fujii group has demonstrated single-cell isolation into microwells using an underlying interdigitated electrode configuration.^19^ Despite the excellent capture efficiency, the microwell closing technique that involves collapsing of an overlying poly(dimethylsiloxane) (PDMS) membrane complicates device operation and can cause mechanical lysis of cells.

Our group has previously reported a DEP-BPE device that has excellent potential for facile single-cell analysis – it is valve-free, expandable, has individual confinements for downstream cell studies and employs immiscible fluid boundaries for reaction unit isolation. The device comprises an array of BPEs aligned to an array of microchambers for cell capture using pDEP, and the captured cells are passively hydrodynamically transferred into the chambers upon disconnecting the AC voltage supply.^5^ Following cell transfer, the chambers need to be sealed-off to avoid cross-contamination during subsequent assays. To accomplish this task, we have demonstrated sealing of the chambers by filling the channels with mineral oil or a hydrophobic ionic liquid to form a phase boundary at the chamber opening. For more robust sealing, we reported a method to generate a solid plug *via* electropolymerization of an ionic liquid at the BPE tip located at the microchamber opening to hold the chamber constituents in place.^20^ Furthermore, we have successfully shown that the DEP-BPE device can be used for lysed-cell assays to uncover cell-to-cell variability^6^ and for isolating circulating tumor cells (CTCs) from patient-derived blood samples.^15^

All the above-mentioned approaches reported by our group employ electrode-based DEP (eDEP) for cell isolation where the tip of a BPE positioned at each chamber opening (micropocket) creates a local electric-field maximum for subsequent cell capture. Despite the excellent micropocket capture (84.4%) and chamber transfer (89.4%) percentages achieved by this approach,^5^ a requirement for precise alignment greatly hinders its practical application. The incorporation of a BPE array or an array of wireless electrodes is a key advancement in our previous work. As the entire BPE array is driven by a single pair of driving electrodes, the array can be facilely expanded as required by the application. For optimum functioning of this DEP-BPE platform, each BPE tip should be aligned with high precision – about 10 μm away from each micropocket opening (5 μm tolerance). Additionally, the pointed shape of the electrode tips is difficult to fabricate reliably and lacks rotational tolerance, which necessitates further alignment accuracy. This precise alignment becomes arduous with increasing array dimensions. Consequently, larger errors are inevitable in massive arrays, and diminishes the robustness of the platform. From a scaling-up standpoint, the requirement for precision and accuracy poses a challenge, given that artifacts caused by factors such as birefringence and shrinkage are prevalent in mass-production methods such as injection molding, thereby altering the landmarks intended for automated device assembly.^21^

Insulator-based DEP (iDEP) is a variant of DEP where local electric field hotspots are generated amongst insulating posts or walls. Importantly in this technique, as the cells do not come in direct contact with the electrodes, the cells are protected from injurious effects caused by electrodes and reactive oxygen species (ROSs).^22,23^ Thus, we identify iDEP as a safer technique and a potential approach to tolerance-forgiving designs. Nonetheless, the iDEP approaches that currently focus on single-cell isolation have their own bottlenecks. The self-digitization dielectrophoretic (SD-DEP) chip reported by Qin and co-workers has an excellent single-cell trapping efficiency (92.7%) in microchambers.^24^ However, the 1D array is directly connected to the driving electrodes and therefore, its expandability is limited. Alternate iDEP platforms which demonstrate the single-cell trapping ability, do not have confinements to perform relevant on-chip analyses.^25,26^ Therefore, robust, and facilely producible devices are in demand that circumvent the current limitations.

Herein, we present a single-cell isolation platform driven by iDEP in a two-dimensional array of microchambers addressed by easy-to-align ‘wireless’ BPEs. The performance of the device was validated using human breast cancer cells (MDA-MB-231). We demonstrate that the influence of the electrode position on the electric field strength within the micropocket employed for cell capture becomes more gradual the further it is recessed, reaching an alignment tolerance of 80 μm. Notably, within this error, the efficiency with which pockets are occupied singly was retained at >90% (among occupied pockets). Further, we show that the use of “bar BPEs” (BPEs shared by all chambers in a row along a channel length) significantly simplifies the alignment procedure while giving nearly infinite horizontal tolerance. This work is significant because it retains the advantages of the DEP-BPE platform for selective single-cell capture and analysis while achieving both exceptional tolerance in both *x*- and *y*-dimensions and a decreased demand for photolithographic resolution in electrode fabrication. These advancements allow for facile device fabrication and assembly and alignment-independent single-cell isolation on an expandable array of electrodes. The robustness of the device also potentiates low-cost mass producibility, which enables broad access to single-cell analysis on chip.

## Theoretical background

DEP is a phenomenon that drives the movement of polarizable particles in an external, non-uniform electric field. Importantly, the technique allows for selective manipulation of particles. Since the first report of DEP manipulation of cells in 1966, a multitude of relevant applications such as cell patterning, separation, characterization, and manipulation have been demonstrated.^27^ In a dielectric particle, charge accumulation occurs at the particle–medium interface (Maxwell–Wagner polarization) when subjected to an electric field. The time-averaged dielectrophoretic force is a result of this frequency-dependent dipole moment interacting with the spatial gradient of the applied electric field. The magnitude of the force generated depends on the dielectric properties of both the particle and the medium as shown in eqn (1), where *r* is the radius of the particle, *ε*_m_ is the permittivity of the medium, Re[*K*(*ω*)] is the real part of the Clausius–Mossotti (CM) factor and *E* is the electric field.

1 *F*_DEP_ = 2π*r*^3^*ε*_m_Re[*K*(*ω*)]∇|*E*|^2^

The CM factor is a representation of the strength of the effective polarization of the particle based on the complex permittivity of the particle 
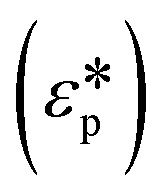
 and that of the medium 
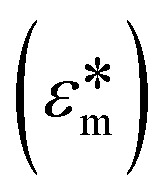
 as shown in eqn (2).2
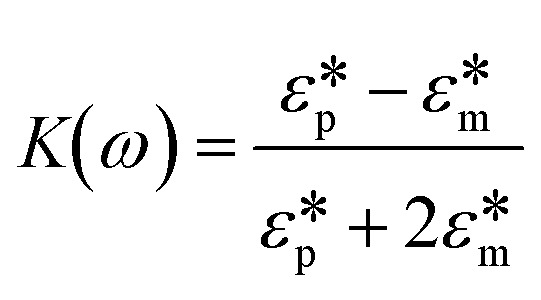
When the particle is more polarizable than the medium, a net DEP force acting on the particle moves it in the direction of increasing electric field strength and is referred to as positive DEP (pDEP). In contrast, when the particle is less polarizable than the medium, it will be displaced towards low electric field points and is referred to as negative DEP (nDEP). The specific frequency at which the response transitions from pDEP to nDEP or *vice versa* is called the crossover frequency (cof) and differs by cell phenotype thereby enabling selective cell separation using DEP.

## Materials and methods

### Chemicals

The silicone elastomer kit (Sylgard 184), Bovine serum albumin (BSA) (Biotech grade) and 0.25% trypsin–EDTA (1×) were obtained from Fisher Scientific (Thermo Fisher Scientific, Inc., Waltham, MA). Pluronic® F-127, dextrose (d-glucose), Tris·HCl stock and DMEM/F12 culture medium were purchased from Sigma-Aldrich, Inc. (St. Louis, MO). Sucrose was purchased from MilliporeSigma (Milwaukee, WI). All solutions were prepared in type 1 water (18.2 Ω cm). The “DEP buffer” was prepared with 8.0% sucrose, 0.3% dextrose and 0.1% BSA in 1.0 mM Tris buffer (conductivity: 60–65 μS cm^−1^).

### Cell culture

Breast adenocarcinoma cell line MDA-MB-231 was purchased from ATCC (Manassas, VA). The cells were cultured in DMEM/F12 medium supplemented with of 10% fetal bovine serum albumin. The cell culture was incubated at 37 °C and 5% CO_2_ and subcultured (passaged) after reaching ∼80% confluency. All cells used in experiments were obtained after 2 days of culturing and were within the first twelve passages to avoid deviation from the parental line in phenotype. To obtain cells for DEP experiments, the cells were first cleaved from the culture flask using 0.25% trypsin–EDTA (1×) and subsequently pelleted by centrifugation at 1100 rpm for 5 min. The cell pellet was washed twice with fresh media by resuspension and centrifugation, and the final cell pellet was resuspended in DEP buffer. Following two washing steps with DEP buffer, the cell concentration in buffer was measured using Countess II, Invitrogen (Waltham, MA) and maintained at 8 × 10^5^ cells per mL for all experiments.

### DEP-cell capture

Prior to DEP-based cell capture, the iDEP-BPE devices were coated with 3.0 μM Pluronic® F-127 overnight at 4 °C. The Pluronic® coated devices were then flushed with fresh DEP buffer for 15 min at a flow rate of 100 nL min^−1^. Subsequently, the cell sample was introduced through the device inlet and a flow stabilization time of 2–3 min was allowed. Both the volumetric flow rate and the magnitude of the applied AC voltage employed for cell capture are reported for each experiment described in the Results and discussion section. The volumetric flow rate was maintained using a Pico Plus Elite syringe pump (Harvard Apparatus, Holliston, MA) coupled with a 500 μL glass syringe (Hamilton Company, Reno, NV) withdrawing the solution at the device outlet. After stabilization, an AC voltage was applied at 100 kHz to the driving electrodes using Tektronix AFG3011C waveform generator (Tektronix, Beaverton, OR) paired to Trek Model 2205 amplifier (Trek, Lockport, NY). The 100 kHz frequency was chosen to obtain a stable and strong pDEP response. A period of 20 min was allowed for cell capture and subsequently, the AC voltage was switched off, allowing the cells to hydrodynamically transfer into the chambers at the same flow rate. The device was imaged using AZ-100 microscope (Nikon, Tokyo, Japan) and images after capture and transfer were obtained.

### Device fabrication

The PDMS monolith with embedded microfluidic channels and chambers was fabricated using standard soft lithography methods. Briefly, a silicon master mold was fabricated using photolithography. First, the 4 inch Si wafer was spin coated with SU-8 2025 negative photoresist (MicroChem Corp., MA) and was soft baked at recommended temperatures. The soft-baked wafer was then exposed to UV light through a photomask (patterned with chromium) (Front Range Photomask, Las Vegas, NV) using a mask aligner (ABM-USA, San Jose, CA). Following standard post-exposure baking procedures, the unwanted photoresist was removed using SU-8 developer (MicroChem Corp., MA) and hard baked to yield the SU-8 master mold. The wafer was used to fabricate the PDMS replica by caste-molding. The conventional 10 : 1 ratio of PDMS elastomer base to curing agent was used and the thoroughly mixed contents were degassed before pouring on the wafer. The PDMS was cured for 72 h at room temperature. The cured PDMS designs were cut separated, and inlets (3.0 mm) and outlets (1.0 mm) were punched using biopsy punches.

The thin-film gold electrodes were also fabricated using photolithography. Commercially available glass slides with 100 nm-thick Au film adhered on 5 nm Cr film (Evaporated Metal Films, Ithaca, NY) were first spin coated with AZP4620 positive photoresist (Integrated Micro Materials (IMM), Argyle, TX) and then subjected to a pre-exposure bake of 1 min at 110 °C. The slides were then UV exposed with a mylar photomask (FineLine Imaging, Colorado Springs, CO). Unwanted photoresist was removed with AZ 400 K developer (IMM). Subsequently, the exposed metal thin film was chemically etched, first with 10% KI/2.5% I_2_ and then chrome etchant (Sigma-Aldrich, St. Louis, MO), rinsing with d.d.i. water between each etching step. The unexposed photoresist was finally removed with acetone. The patterned-gold electrodes were then cleaned with 200 proof ethanol (Decon Labs, King of Prussia, PA) and were dried with a stream of N_2_ gas. Prior to device assembly, the patterned slides were cleaned in a base bath of 1 : 1 : 1 ammonium hydroxide, hydrogen peroxide and type 1 water (“basic piranha”) at 95 °C for at least 15 min.

The PDMS monolith and the glass slide were then thoroughly cleaned with 200 proof ethanol and were dried with N_2_. Subsequently, they were plasma treated (Harrick Plasma, Ithaca, NY) at high power for 30 s for surface activation. The plasma treated PDMS monolith was aligned with the patterned electrodes under a microscope to yield a micropocket-to-electrode gap at the magnitude indicated for each experiment in the Results and discussion section. The alignment was facilitated by adding a few drops of ethanol between the monolith and substrate, and after alignment, the devices were placed in an oven at 65 °C overnight to drive off the ethanol and to ensure irreversible bonding.

### Device geometry and dimensions

The PDMS design consists of four main parallel channels each with a width of 0.1 mm and a length of 6.69 mm, connected to a common inlet and an outlet *via* bifurcated channels. Each parallel channel contains 20 microchambers (each 200 μm wide × 400 μm long) aligned on either side of the channel along the length, leading to a total of 80 chambers. Each chamber is connected to the main channel *via* a micropocket. While the width of the micropocket was kept constant at 20 μm, the depth (distance perpendicular to the channel axis) was altered (25, 20, 15 μm) to evaluate its impact on single-cell capture. The chamber, micropocket and the leak channel shared a common height of 25 μm. This design was adopted from previous work^5^ and all other design features including the length, width and the position of the leak channel, inter-channel distance and inter-chamber distance were kept constant.

Two electrode formats were evaluated in this study. The “paddle-shaped” electrodes were 100 μm wide and the length was varied appropriately to maintain the desired micropocket-to-electrode gaps. The “bar electrodes” were approximately 0.5 mm wide (adjusted to have precise micropocket-to-electrode gaps) and 6.12 mm long so that an entire row of chambers were aligned to a single shared electrode, instead of addressing each column of chambers with separate electrodes. Both types of electrodes were addressed using a single pair of driving electrodes, connected to external wire leads.

## Results and discussion

**Fig. 1:**
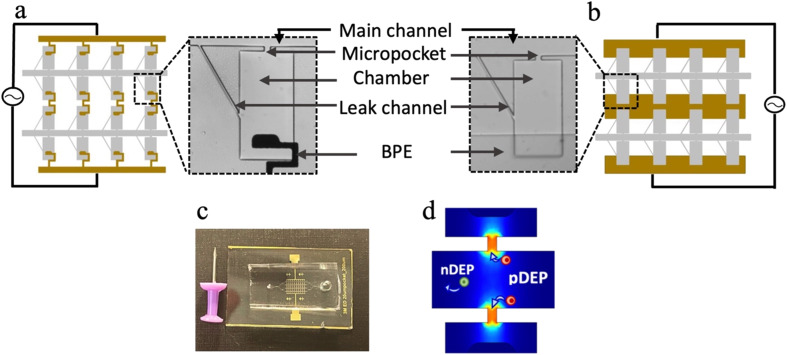
(a) Illustration of a section of “paddle” BPE array aligned with the 2D array of chambers with AC voltage applied and labeled brightfield micrograph of an individual array element aligned to a paddle BPE. (b) Illustration of a section of “bar” BPE array aligned with the 2D array of chambers with AC voltage applied along with the enlarged individual array element. (c) Assembled iDEP-BPE device along with a drawing pin for size comparison. (d) Schematic representation of polarizable cells showing pDEP and nDEP responses.

BPEs are wireless electrodes that are energized by a single pair of leads and thus enable facile expansion of arrays as needed. The work presented here utilizes two distinct BPE geometries, *i.e.*, paddle and bar. These BPEs are aligned to a two-dimensional array of microchambers as shown in Fig. 1a and b. Each chamber comprises a micropocket that connects the chamber to the main channel, a leak channel to provide sufficient drag force to facilitate the hydrodynamic transfer of the captured cell into the chamber and an in-chamber BPE that is aligned at a specified distance from the inner edge of the micropocket. Upon assembly, the iDEP-BPE device shown in Fig. 1c is obtained. In contrast to our previous eDEP platform,^5^ in the iDEP-BPE device, the insulating PDMS side walls of the micropocket create a constriction and a consequent electric field “hot spot” (local maximum) within the micropocket as shown in Fig. 1d.

This combination of iDEP with the DEP-BPE platform provides a critical advantage – the electrode can be stationed entirely within the chamber, farther away from the micropocket, while preserving the capability of selective and individual-cell isolation observed in eDEP. Further, the electrode shape becomes less critical and high-aspect-ratio features or sharp angles are not required. Importantly, the iDEP mechanism prevents the direct contact of cells with the electrodes and the resulting detrimental impact of electrodes and ROSs on cells thus preserving the cell viability.^22,23^ Therefore, iDEP is potentially beneficial as the driving concept for tolerance-forgiving designs needed for mass production.

### Recessed electrodes increase alignment tolerance

The primary objective of this study is to increase the translational and rotational alignment tolerance of this DEP platform. Therefore, we first investigated the effect of the *y*-dimensional position (the direction perpendicular to the channel axis) of the BPE on the strength of the electric field within the micropocket. A wide, paddle-shaped electrode was positioned within the chamber at distinct distances of 0, 15, 25 and 35 μm away from the inner edge of the micropocket. We hypothesized that a wide electrode geometry that occupies a considerable area of the chamber would increase rotational tolerance.

**Fig. 2:**
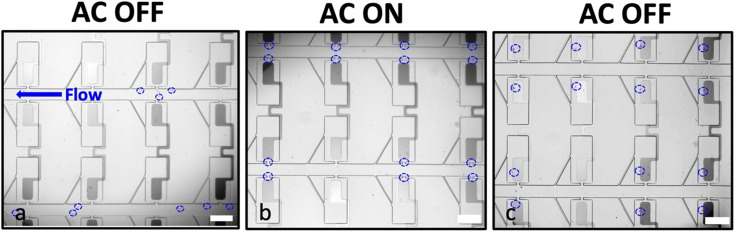
Brightfield micrographs showing the sequential steps of cell capture and transfer. (a) Pressure driven flow carries MDA-MB-231 cells (blue dashed circles) along the main channel (right to left) prior to voltage application. (b) Single-cell capture by pDEP under an AC voltage (30 V_p–p_, 100 kHz) applied at the driving electrodes. (c) Hydrodynamic transfer of cells into the chambers upon turning off the voltage. Scale bar, 200 μm.

Fig. 2 shows the general workflow followed in all the experiments. In a typical experiment, the iDEP-BPE device was first positioned on the microscope stage as shown in Fig. S1† and was flushed with DEP buffer (60–65 μS cm^−1^) for at least 15 min. Second, the MDA-MB-231 breast cancer cells of confirmed viability and adjusted concentration (8 × 10^5^ cells per mL) in DEP buffer were introduced into the device inlet, and the desired flow rate was established. Table S1† shows the viability, average cell diameter and concentration of flowed-through cells in three separate experiments as quantified by the Countess™ system. Movie S1† shows the MDA-MB-231 cells stained with calcein AM flowing in the main channel as further evidence of flowed-through cells being viable. As illustrated in Fig. 2a, in the absence of an applied voltage, the cells transit the parallel channels aided by pressure-driven flow. The cells do not enter the capture pockets or chambers in the absence of an applied voltage. Third, an AC voltage at pre-defined frequency was applied at the driving electrodes to attract cells to the micropockets by pDEP (Fig. 2b). The crossover frequency for MDA-MB-231 cells is reported to be 20–50 kHz,^28^ and therefore, a frequency of 100 kHz was chosen to ensure that all viable cells uniformly experienced a strong pDEP force. We have previously shown that the voltage and flow rate employed in the DEP-BPE device can be independently optimized to balance their effects thereby assisting single-cell capture.^5^ Accordingly in preliminary work, an optimum voltage of 30 V_p–p_ and a flow rate of 200 nL min^−1^ (average linear velocity: 330 μm s^−1^) were employed. Fourth and finally, following dielectrophoretic capture, the voltage was switched off to allow the hydrodynamic transfer of cells into the chamber as shown in Fig. 2c.

**Fig. 3:**
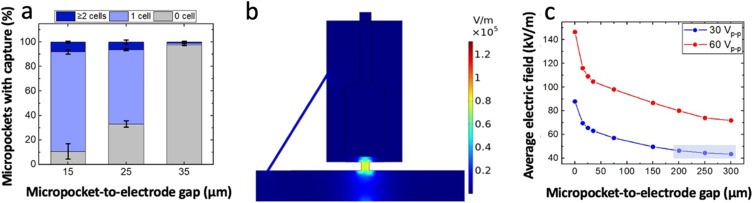
(a) Bar graph showing the percentage of empty, singly, and multiply occupied pockets as a function of micropocket-to-electrode-gap size. Cell concentration of 8 × 10^5^ cells per mL, 200 nL min^−1^ flow rate, applied voltage of 30 V_p–p_ at 100 kHz. (b) Surface plot of the result of a numerical simulation of the electric field strength in a single array element. Micropocket-to-electrode gap 15 μm, 30 V_p–p_. (c) Plot of the electric field strength, averaged over the area of the micropocket, as a function of the micropocket-electrode gap. Applied voltage, 30 V_p–p_ and 60 V_p–p_ at 100 kHz.

Using the conditions described above, the impact of the size of the electrode-pocket gap (0, 15, 25 and 35 μm) was evaluated. The results indicate that a 0 μm gap facilitates multi-cell capture (Fig. S2†) followed by continuous pushing of cells onto the electrode as there is no space for an electric field minimum (trough) between the micropocket and the electrode. However, when the front edge of the electrode was recessed by 15 μm, an excellent single-cell micropocket occupancy (percent of the total micropockets occupied) of 81% was observed (Fig. 3a). We expected the single-cell occupancy to change most significantly when the gap size changed from 0 μm to 15 μm, and for the electrode-position effect to decrease beyond 15 μm. However, for gaps of 25 μm and 35 μm, we observed a sharp decline in percent micropocket occupancy, with values dropping to 65% at 25 μm and just 1% at 35 μm. Moreover, as shown in Fig. 3a, the number of empty micropockets significantly increased suggesting that the electric field in the micropockets was too weak to accomplish cell capture. These results indicate that a gap size of 0 to 35 μm is inappropriate for a robust single-cell isolation platform because a minute error in alignment significantly impacts the performance. Therefore, to further investigate the suitable gap sizes, numerical simulations were employed.

To perform numerical simulations COMSOL Multiphysics 5.2a was used and the electric field strength was simulated for distinct gap sizes including the experimentally tested configurations. All simulations were performed at an applied voltage of 30 V_p–p_ and a frequency of 100 kHz. The results confirmed the presence of an electric field maximum within the micropocket as shown in Fig. 3b. As depicted in Fig. 3c, the average electric field within the micropocket was found to exponentially decay with increasing micropocket-to-electrode distance. The steep decay observed at short distances supported our experimental observations in 15- to 35 μm gap assemblies. Further, it was observed that at 200 to 300 μm gaps, the slope was flattened. Based on this observation, it can be claimed that, as the front edge of the electrode is positioned increasingly farther into the chamber, the rate of decrease in electric field strength within the micropocket becomes more gradual. This result is significant because the gradual slope is promising for platforms with high tolerance.

Therefore, the applied voltage was scaled up (to 60 V_p–p_) to yield a sufficient electric field strength in the micropocket for cell capture. Since the highest single-cell occupancy in micropockets was experimentally observed at 30 V_p–p_ at a gap size of 15 μm, numerical simulations were carried out for 200, 250, 300 μm-gap assemblies where the applied voltage was adjusted to match the average electric field strength observed at 15 μm. Table 1 shows the voltage that would provide matched average electric field strengths in 200 to 300 μm-gap assemblies as compared to a 15 μm-gap assembly. Accordingly, an applied voltage of 60 V_p–p_ was estimated to be sufficient for 200 to 300 μm-gap assembles. A key point is that at this doubled voltage the flattened slopes were maintained for 200- to 300 μm gaps confirming that the observed result is strictly dependent on the gap size. This voltage was further optimized experimentally as shown in Fig. S3.† Also, the flow rate was re-optimized for the gap size and the applied voltage. Consequently, an optimum voltage of 66.2 V_p–p_ and a flow rate of 90 nL min^−1^ (average linear velocity: 200 μm s^−1^) was employed in the experiments that follow. As the electric field distribution is distinct from that employed in our previous cell isolation work,^5,6,15,29^ the viability of cells during the capture time and post-transfer was qualitatively assessed using Calcein AM, a cell-permeant dye that renders viable cells fluorescent through the action of intracellular esterases. Fig. S4† shows the brightfield and fluorescence micrographs of cells captured at 66.2 V_p–p_ and held over a period of 20 min and post-transfer. As shown in a plot of the intensities of two cells over 20 min (Fig. S4k†), the cells are highly fluorescent indicating viability, and the fluorescence intensity is maintained over the entire time, indicating intactness. Moreover, it is important to highlight that non-viable cells exhibit minimal dielectrophoretic mobility which is attributed to the changes in the electrical properties of cells upon damaging membrane integrity or due to electroporation. Further, we have observed that at higher applied voltages (∼95 V_p–p_), the cells respond to pDEP, but in less than 20 min, lose the defined membrane boundary followed by their escape from the micropocket (loss of DEP response). To provide a quantitative aspect to the viability of captured and transferred cells, the fraction of fluorescent cells was counted, and 100% of the captured and transferred cells appear significantly fluorescent. To further validate the pDEP response of cells that were held under strong electric fields, after 20 min of voltage application at 66.2 V_p–p_, power was turned off and back on again to see if cells responded similarly as before. Movie S2† show the cells repeatedly responding to the electric field even after being held at high electric field for 20 min, thus confirming their intactness.

**Table 1:** Average simulated electric field strengths over the area of the micropocket calculated using numerical simulations for devices with 200- to 390 μm gaps as compared to a 15 μm-gap device

Micropocket-to-electrode gap (μm)	Voltage (V_p–p_)	EF_Avg_ (kV m^−1^)
15	30	69.5
200	60	80.0
250	60	74.0
300	60	71.8
330	60	67.5
370	60	63.5
390	60	62.7

### An intermediate micropocket depth maximizes the single-cell isolation efficiency

The micropocket depth (the dimension perpendicular to the channel axis) is a feature that could immensely contribute to the single-cell capture in the iDEP-BPE device. As compared to our reported eDEP work where the sharp electrode tip defined the local electric field maximum, in the iDEP-BPE device, the electric field is augmented within the entire micropocket. Consequently, this space facilitates the formation of pearl chains (strings or clusters of polarized cells) which are also shielded from the flow by the micropocket walls. This scenario is highly undesirable for platforms intended for single-cell isolation and thus, was addressed by optimizing the micropocket dimensions.

**Fig. 4:**
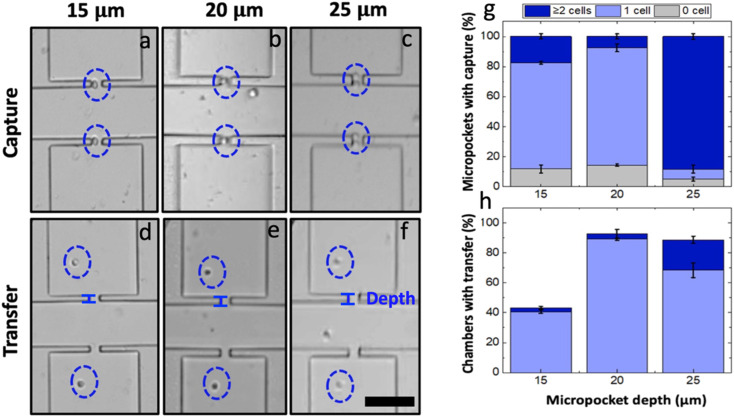
Brightfield micrographs of (a–c) cell capture and (d–f) hydrodynamic cell transfer in devices with three distinct micropocket depths: 15, 20, 25 μm, respectively at 66.2 V_p–p_ and 90 nL min^−1^. Bar graph showing the (g) percentage of empty, singly, and multiply occupied pockets as a function of micropocket depth. (h) Of the occupied pockets, the percentage of singly and multiply transferred cells. Scale bar, 100 μm.

Accordingly, the effect of the micropocket depth on the single-cell capture in the iDEP-BPE device was studied. In a device with a 300 μm electrode-pocket gap, three distinct micropocket depths, *i.e.*, 15, 20 and 25 μm, were tested using the optimized voltage (66.2 V_p–p_) and flow rate (90 nL min^−1^) conditions at a cell concentration of 8 × 10^5^ cells per mL in DEP buffer. The results are illustrated by exemplary brightfield micrographs obtained following cell capture (Fig. 4a–c) and transfer (Fig. 4d–f) as well as plots of the percent of total pockets at which zero, one, or multiple cells were captured (Fig. 4g) or percent of total chambers with one or multiple cells transferred (Fig. 4h) in devices with each of these three pocket depths. Pockets with 15 and 20 μm depths yielded single-cell occupancies of 70 and 77%, respectively. Meanwhile, in the 25 μm-deep pockets, only 8% of the pockets were singly occupied while 90% of the pockets were multiply occupied. However, during the hydrodynamic transfer, 68% of the captured cells were lost from the 15 μm deep pockets. This loss occurs because the captured cells are partially exposed to the main-channel flow (average cell diameter 18.8 ± 1.4 μm) resulting in pulling away of cells by lift force into the main channel upon turning the voltage off.^5^ Confirming this hypothesis, out of multiply occupied pockets in 25 μm-deep pockets, only 20% were multiply transferred while 70% were singly transferred (*i.e.*, the cell nearest the channel was pulled back into the channel). In contrast, a 90% single-cell transfer was observed in 20 μm-deep pockets. Based on these results, it can be claimed that an intermediate micropocket depth of 20 μm maximizes the percent single-cell occupancy to be 77% in the iDEP-BPE device as studied for MDA-MB-231 cells. It is also worth noting that in all the pocket dimensions, the overall percent micropocket occupancy was above 90%. Therefore, this result is significant and suggests that, for smaller cells, 15 μm-deep pockets can be used while for larger cells, 25 μm-deep pockets can be used while maintaining constant capture conditions.

### The iDEP–paddle-BPE device offers a vertical alignment tolerance of 80 μm

To validate the robustness of the iDEP–paddle-BPE device, cell capture experiments were performed under the optimized flow rate, voltage and micropocket depth conditions at distinct gap sizes of 200, 250 and 300 μm. While single cells are important for revealing cellular heterogeneity, in the realm of rare cells, even isolation of a small group of cells (2–3 cells) can be beneficial compared to other techniques that derive traits of cells in ensemble. However, since the prime focus of the iDEP-BPE platform is single-cell isolation, the alignment tolerance was defined based on the single-cell occupancy. Accordingly, 75% single-cell occupancy observed at a micropocket-to-electrode gap of 300 μm was set as the threshold and the micropocket-to-electrode alignments that yield single-cell occupancies that are not significantly different from the set threshold were considered “tolerant”.

**Fig. 5:**
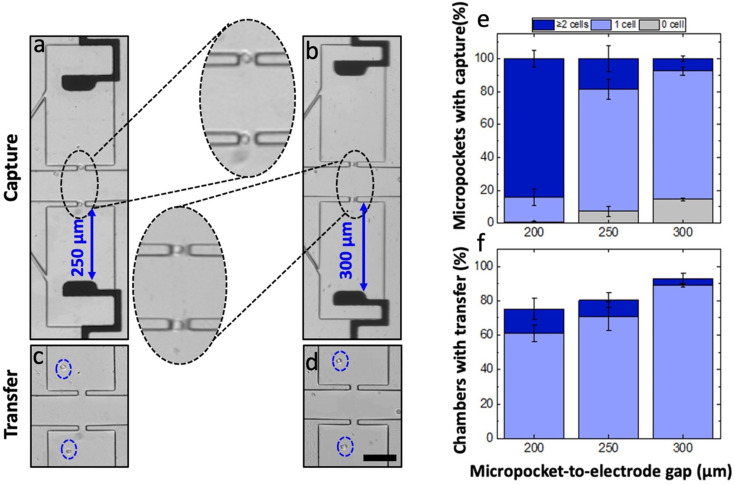
(a and b) Brightfield micrographs of cell capture and (c and d) Brightfield micrographs of hydrodynamic cell transfer in large-gap assemblies: 250 and 300 μm, respectively for 20 μm deep pockets at 66.2 V_p–p_ at 90 nL min^−1^. (e) Bar graph showing the percentage of empty, singly and multiply occupied pockets as a function of pocket-electrode gap. (f) Of the occupied pockets, the percentage of singly and multiply transferred cells as a function of pocket-electrode gap. Scale bar, 100 μm.

Fig. 5 shows exemplary brightfield micrographs and plots of the percentage of chambers occupied by zero, one, or multiple cells for each gap size following each the capture and transfer steps. Although a closely similar performance was expected in all three gap sizes based on the numerical simulation results (Fig. 3c), the 200 μm-gap assembly was found to significantly deviate from the other two gap sizes. For the 200 μm gap, 84 ± 5% of the pockets were multiply occupied (out of those occupied pockets, 60% of the chambers showed single-cell transfers) (Fig. 5e and f and S5†). This multiple occupancy suggested that at 200 μm, the electric field strength in the pocket is too high. As shown in Fig. 5e and f, 250- and 300 μm gap assemblies showed similar performances to each other, and excellent single-cell occupancies of 74 ± 6% and 78 ± 2% respectively, were observed. Moreover, out of the captured cells, 71 ± 8% and 89 ± 1% were singly transferred thereby confirming the excellent capture and transfer conduct of the iDEP-BPE platform regardless of a 50 μm alignment difference in the *y*-dimension.

**Fig. 6:**
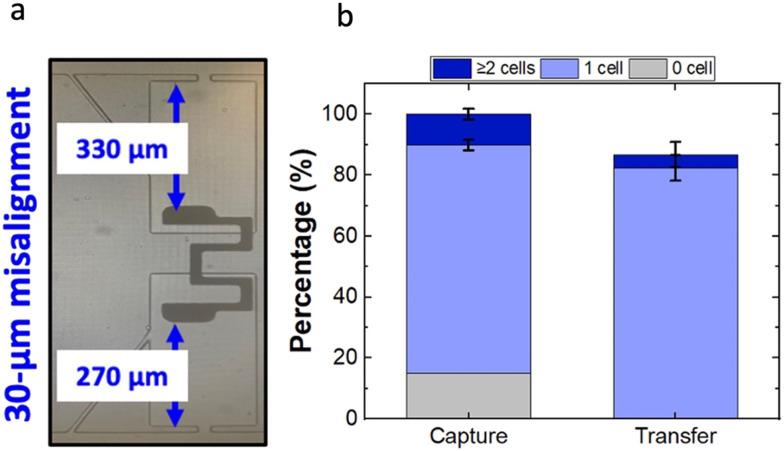
(a) Brightfield micrograph of a purposely misaligned PDMS–electrode assembly with pocket-electrode gap measured with corresponding microscope settings. (b) Bar graph showing percentage of empty, singly, and multiply occupied pockets and singly and multiply occupied chambers out of occupied pockets. Cell concentration of 8 × 10^5^ cells per mL, 90 nL min^−1^ flow rate, applied voltage of 66.2 V_p–p_ at 100 kHz.

While the 200- to 300 μm gaps were fabricated using distinct photomask designs, an alternative 330 μm gap was created by purposely misaligning the array, to check if the capture/transfer performances are sustained beyond a 300 μm gap size. Since the electrodes are wireless and a single row of electrodes is shared by two rows of chambers from either side, the resulting device featured 40 chambers with a micropocket-to-electrode gap of 270 μm while the other 40 had a gap of 330 μm. To quantify the micropocket/chamber occupancy, the half of the device featuring a 330 μm gap was used. The capture and transfer performance of the misaligned device is shown in Fig. S6.† As depicted in Fig. 6b, the results indicated that the device still retains a 75% single-cell occupancy in micropockets (capture) and an 82% single-cell chamber occupancy (transfer). Importantly, as calculated for the 40 chambers with 330 μm gap, 81% of the pockets were singly occupied and 76% of those cells was singly transferred. This result is significant because the performance is not significantly impacted by the misalignment. Fig. S7† shows the statistical comparison of single-cell occupancies at the experimentally tested gap sizes. Accordingly, it was observed that there is no significant difference between the mean single-cell occupancies at 250 μm or 330 μm gaps as compared to a 300 μm gap. Further, the single-cell occupancies at 250- and 330 μm gaps were also not significantly different, thus making the entire 80 μm distance tolerant.

Therefore, an additional 30 μm (up to 330 μm) tolerance is added on top of the previously established 50 μm alignment tolerance. Therefore, it can be concluded that our iDEP-BPE device has a vertical tolerance of at least 80 μm. As predicted by the numerical simulations, we anticipate that the electrode can be moved farther inwards thereby providing additional alignment tolerance. Nevertheless, as the depth of the current chamber is 400 μm, the maximum distance that the electrode could be practically recessed is 390 μm leaving the front-most 10 μm of the electrode to be within the chamber. And in this work, we have successfully demonstrated the functionality up to 330 μm. Further, as depicted in Fig. 3c (at an applied voltage of 60 V_p–p_) the average electric field within the pocket drops by 72 kV m^−1^ as the electrode is increasingly recessed within the first 250 μm of the chamber length. However, from 250 to 390 μm, the additional drop is only 12 kV m^−1^. Across the range of gap sizes that we have experimentally confirmed to be tolerant (250–330 μm), the drop is 6.5 kV m^−1^. In other words, for a 250 μm gap, the electric field within the micropocket is only 9.5% higher compared to a 330 μm gap. Simulation shows that even further recessing the electrode (330–390 μm) decreases the electric field strength in the pocket by an additional 7.1% (16.6% total). If the applied voltage is optimized for a gap of 330 μm (about the middle of these two ranges), it is anticipated that misalignments to 250 μm (+9.5%) and 390 μm (−7.1%) would be equally tolerated. It is worth noting that while the manufacturing precision critically boosts the device functionality, such precision inevitably heightens the production cost. The reported low-cost, adaptable mechanical jigs and automated aligners, particularly for soft lithography, have accuracies 50–100 μm cm^−1^ and 50 μm cm^−1^,^30^ respectively. Since a fully expanded DEP-BPE array^29^ is also in the order of 1 cm, a tolerance of 80 μm is a significant improvement in the standpoint of cost-effective device alignment. Additionally, the extreme requirements of shape conformities further escalates the cost. Therefore, this study demonstrates significant potential for the cost-effective, large-scale fabrication of microfluidic devices designed for single-cell analysis.

To explicitly characterize the iDEP-BPE device, numerical simulations were performed to yield distributions of the electric field strength and pDEP force across the four parallel channels.^29^ As shown in Fig. S8a,† for a 300 μm-gap assembly, the electric field strength was found to be slightly higher in the top-most and bottom-most micropocket in the cutline, a result which is consistent with our previous work.^29^ The enhanced multi-cell capture in the leading chambers is attributed to this distribution. Further, it was observed that the pDEP force calculated using eqn (1) varied as shown in Fig. S8b.† However, the observed force is well above the minimum force required (as dictated by the Stroke's drag equation) to pull a cell into the micropocket. Similarly, Fig. S8c and d† depict the variation of electric-field strength and pDEP force, respectively, in a 330 μm-gap assembly along a cutline through the micropockets of four parallel channels.

### The iDEP–bar-BPE device offers two-dimensional alignment tolerance

**Fig. 7:**
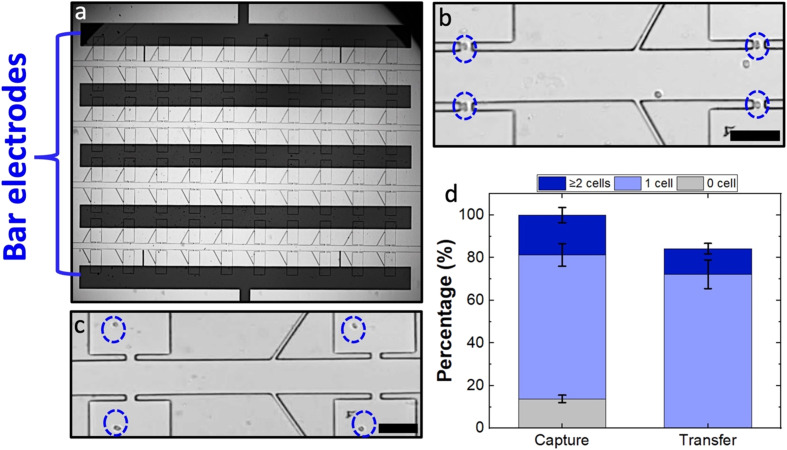
Brightfield micrograph showing (a) bar electrodes aligned to the 2D array of chambers at a micropocket-to-electrode gap of 300 μm. (b) cell capture and (c) hydrodynamic cell transfer in the iDEP–bar-BPE device. (d) Bar graph showing percentage of empty, singly, and multiply occupied pockets and singly and multiply occupied chambers out of captured. Cell concentration of 8 × 10^5^ cells per mL, 90 nL min^−1^ flow rate, applied voltage of 66.2 V_p–p_ at 100 kHz. Scale bar, 100 μm.

The position of the electrode along the *x*-direction is also critical. Although the paddle BPEs yield a more robust platform compared to sharp-tip BPEs, the *x*-dimensional alignment error that could be afforded is limited by the width of the paddle electrode, which is 100 μm for the current design. Fig. S9† demonstrates the capture and transfer performances in horizontally misaligned paddle BPEs as compared to a perfectly aligned paddle. As observed in the results, by shifting the paddle BPE 100 μm horizontally, the percent of empty pockets has increased from 15% to 57%. By further increasing the horizontal shift to 150 μm, the percent of empty pockets increased to 73%. These results suggest that the electric field in the micropocket becomes significantly lower even with horizontal misalignments. Furthermore, it is noteworthy that in both the misalignments presented, the majority of the electric field is contributed by the connecting arm (wire lead) which is now positioned within the chamber. However, if the misalignment happened in the *negative x-direction*, electric field strength within the pocket would drop more precipitously and become negligible given a 150 μm shift. Therefore, to address this limitation, we employed a bar-shaped BPE geometry in place of paddle BPEs. As shown in Fig. 7a, the iDEP–bar-BPE device featured five electrodes, each aligned to two adjacent rows of chambers. The performance of this device was evaluated using the previously demonstrated optimum conditions at a micropocket-to-electrode gap of 300 μm. As presented in Fig. 7b and c, it was observed that despite the new geometry of the BPE, the device performed similarly. As per the results, 76% of the micropockets were singly occupied and out of these captured cells, 82% were singly transferred (Fig. 7d). Thus, we have successfully demonstrated that the iDEP–bar-BPE device performs as well as the iDEP–paddle-BPE device. This result is critically important because the bar electrodes have nearly infinite *x*-dimensional tolerance. Finally, we previously demonstrated the applicability of the DEP-BPE platform to selective capture of distinct cell types (breast cancer and melanoma cells including cell lines, patient-derived xenografts, and clinical samples)^5,6,15,29,31^ given the appropriate frequency, and the reported iDEP-BPE device and its increased tolerance is expected to be generalizable.

## Conclusion

In this work, we have presented an iDEP-BPE device for selective single-cell capture and isolation for subsequent on-chip analysis. Relative to the previously reported DEP-BPE device, this iDEP version is suitable for low-cost mass manufacture. Specifically, the iDEP-BPE device has an order of magnitude greater alignment tolerance and much lower demand for spatial resolution in electrode fabrication. The impact of these increased tolerances becomes greater as the array size is scaled, because they mitigate rotational misalignment and lithographic non-uniformities. As an additional benefit, the detrimental impact of electrodes and ROSs is alleviated in the iDEP system, due to a lack of contact between cells and electrodes. Founded on numerical simulations and supported by single-cell isolation experiments, we claim that by placing the electrode farther inwards into the chamber, the alignment-dependent variability in the electric field strength within the micropocket can be decreased. We also show that the micropocket depth can be leveraged to enhance the single-cell occupancy. Further, it was verified that the presented device has a vertical tolerance of 80 μm. And, by integrating with a bar electrode geometry, the device attained nearly infinite horizontal alignment tolerance. The research presented here is of significant importance for the mass production of robust, high-performance microfluidic devices for single-cell studies, advancing beyond laboratory prototyping. We envision the successful commercial manufacture of such devices upon integration with appropriate materials.

## Data availability

A subset of data may be included as ESI† within the manuscript. For further data access, please contact the corresponding author.

## Conflicts of interest

The authors declare no conflict of interest.

## Supplementary Material

LC-025-D4LC00976B-s001
