# Supplementary material for: iDEP-based single-cell isolation in a two-dimensional array of chambers addressed by easy-to-align wireless electrodes
Source: Lab Chip. 2025 Feb 14;25(6):1600–10. doi: 10.1039/d4lc00976b (PMC11826382; doi:10.1039/d4lc00976b)
Supplement: LC-025-D4LC00976B-s001 [file LC-025-D4LC00976B-s001.pdf]

Supplementary Information (SI) for Lab on a Chip.
This journal is © The Royal Society of Chemistry 2025

Supporting Information

iDEP-based single-cell isolation in a two-dimensional array of chambers addressed by easy-to-align wireless electrodes

Thilini N. Rathnaweera and Robbyn K. Anand

Department of Chemistry, Iowa State University, Ames, Iowa 50011, United States

Supporting Information

This PDF file includes:

Table S1
Figures S1 to S9

Table S1. The cell viability, average cell diameter and cell concentration of flowed-through MDA-MB-231 cells as measured by Countess™ in three independent experiments.

Cell concentration (mL^{-1})	Average cell diameter (µm)	Viability (%)
8.21×10^5	17.83	94
9.85×10^5	18.60	92
9.79×10^5	16.56	86

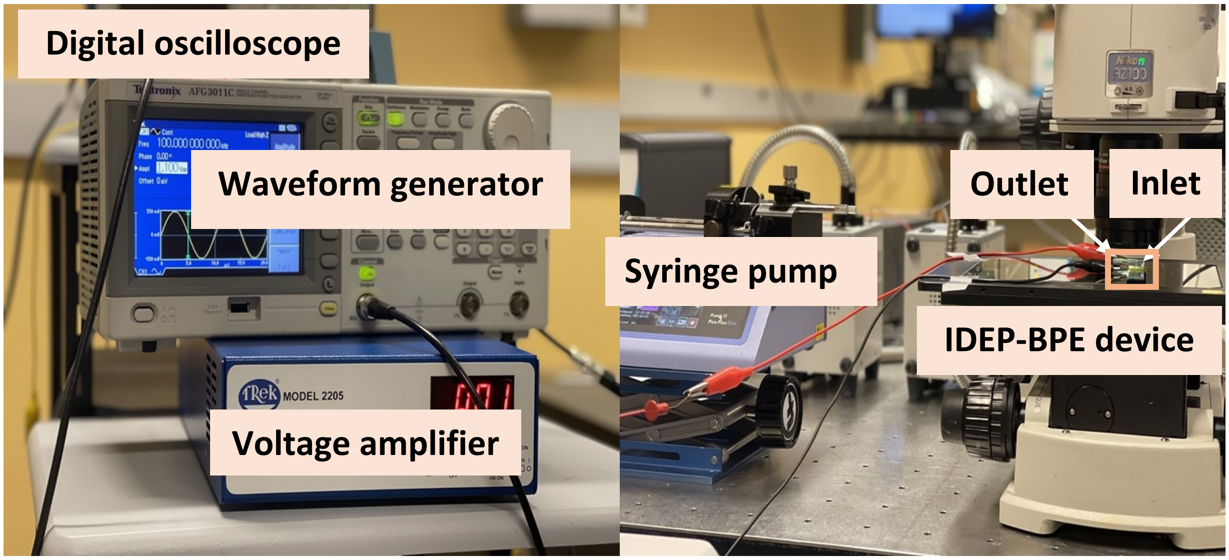

Figure S1. Experimental set-up showing the microfluidic device fastened onto the microscope stage with outlet attached to a syringe pump and the lead electrodes of the bipolar electrode array supplied with high voltage output of the amplifier.

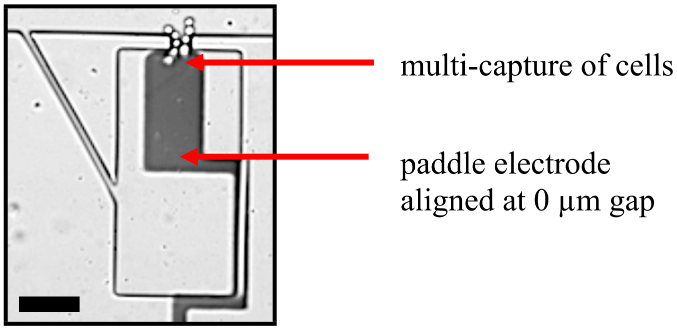

Figure S2. The multi-cell capture and continuous-inward pushing of captured cells in a 0-µm gap assembly. Scale bar, 100 µm

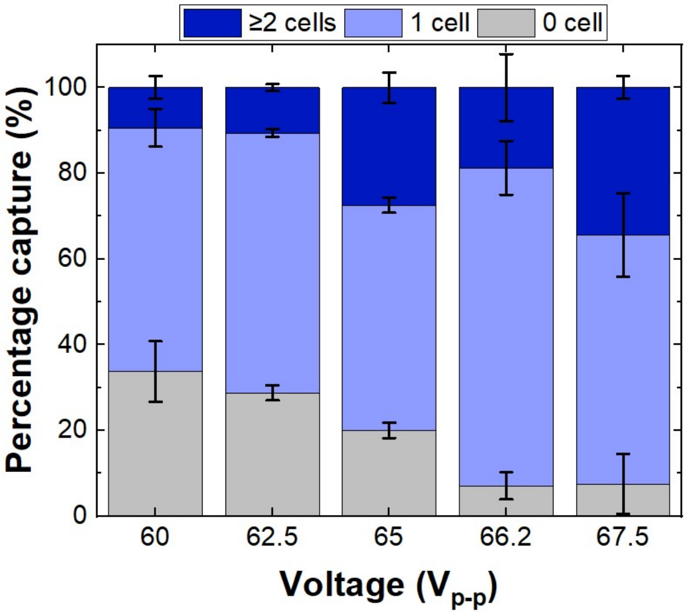

Figure S3. Bar graph showing the percentage of empty, singly, and multiply occupied chambers in a device with 20-μm deep pockets and a 250 μm gap at five distinct applied voltages: 60, 62.5, 65, 66.2, 67.5 $V_{p\text{-}p}$. Cell concentration, 8 $\times 10^5$ cells/mL and flow rate, 90 nL/min.

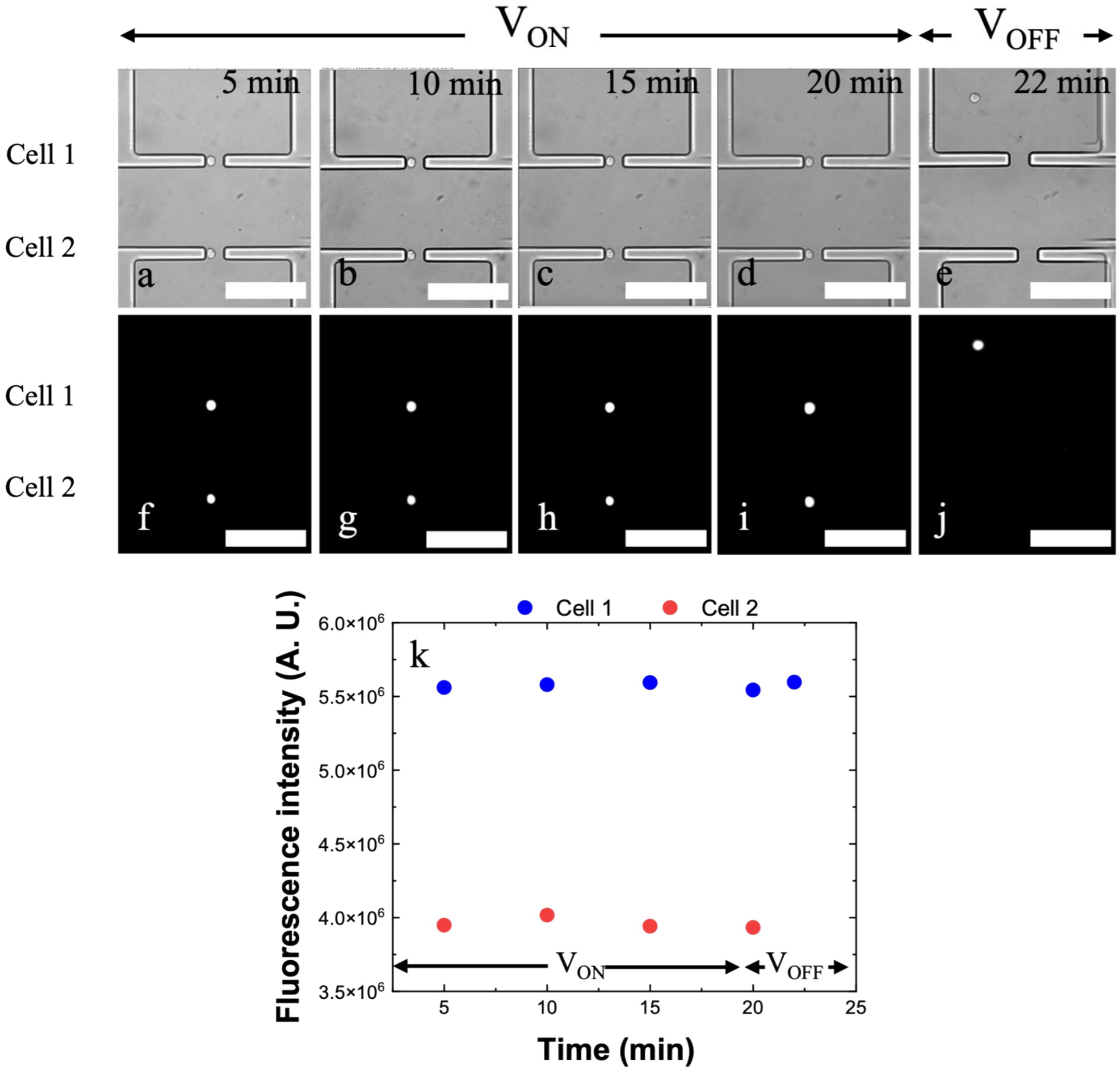

Figure S4. Brightfield (a-d) and fluorescence (f-i) micrographs showing the viability of cells during the 20 min of voltage application (at 66.2 V_{p-p}) and brightfield and fluorescence micrographs (e and j, respectively) showing the cells post-transfer. Cell concentration, 8×10^5 cells/mL, flow rate 90 nL/min. Scale bar, 100 μm

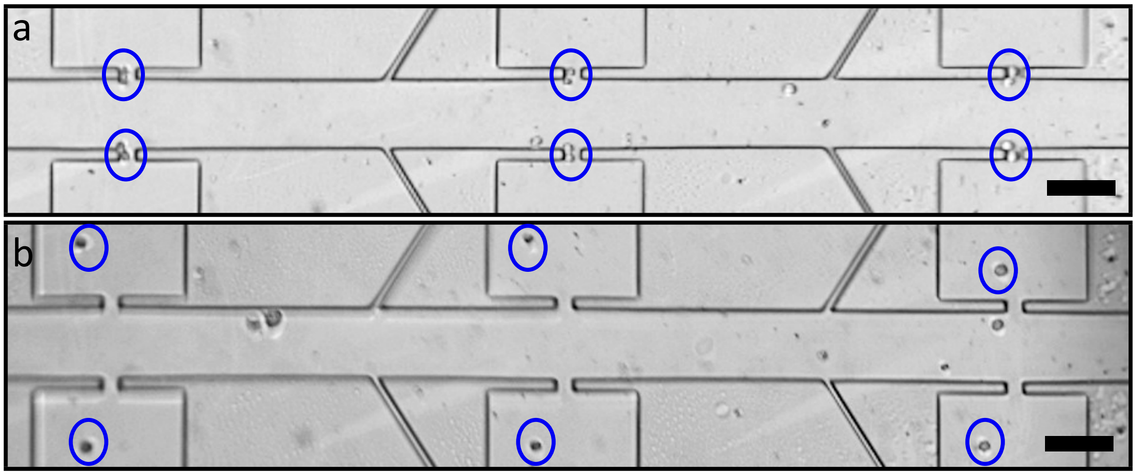

Figure S5. Brightfield images showing (a) capture and (b) transfer in a 200-μm-gapped assembly. 20-μm deep pockets. Cell concentration, 8 $\times 10^5$ cells/mL, flow rate, 90 nL/min at an applied voltage of 66.2 $V_{p\text{-}p}$. Scale bar, 100 μm.

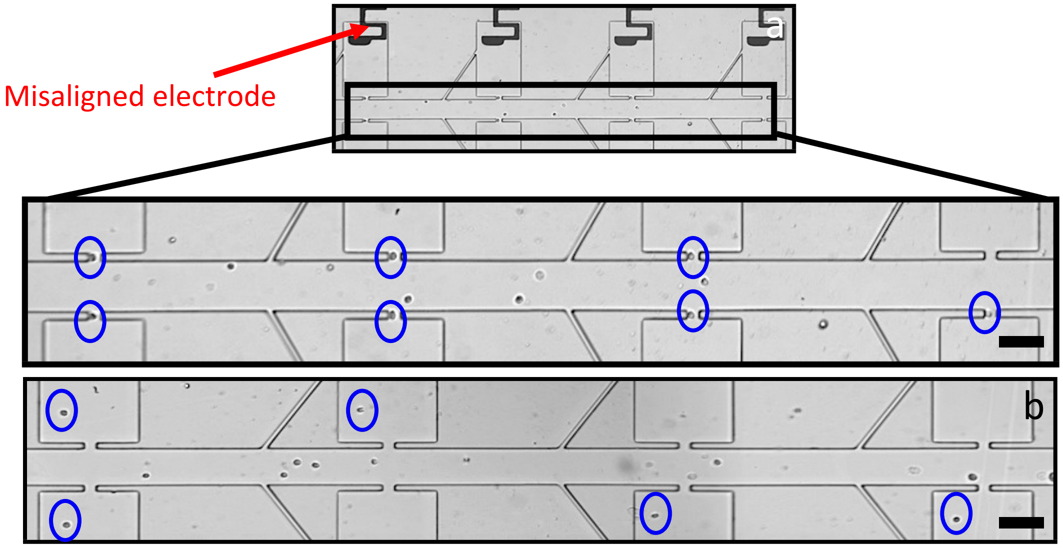

Figure S6. Brightfield micrograph showing (a) cell capture and (b) cell transfer in the misaligned device. Scale bar, 100 μm.

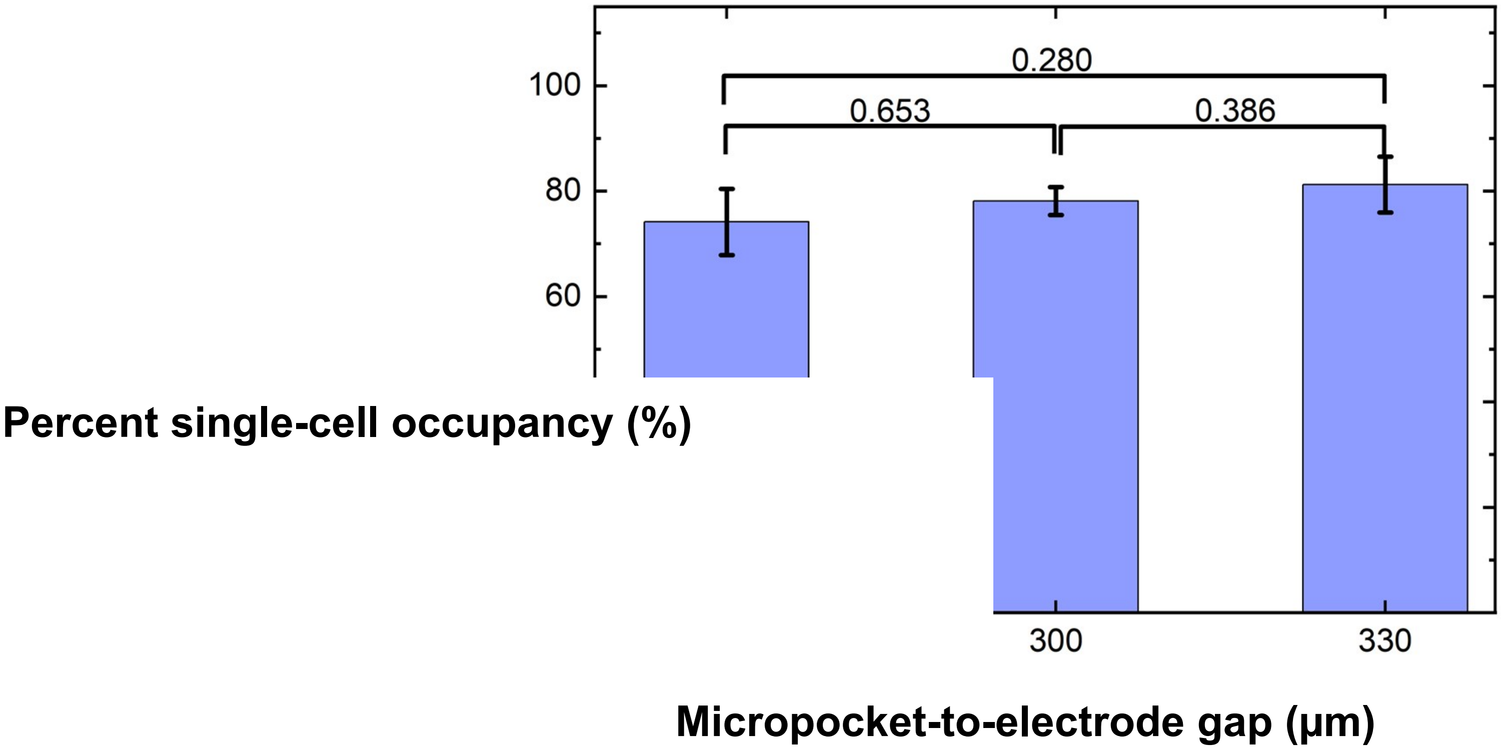

Figure S7. Bar graph showing the percent single-cell occupancies at 250- and 330-µm gaps as compared to the threshold 75% single-cell occupancy observed at a 300-µm gap. The statistical analysis was performed with a two-tailed t-test with unequal variance. Significance determined at α = 0.05.

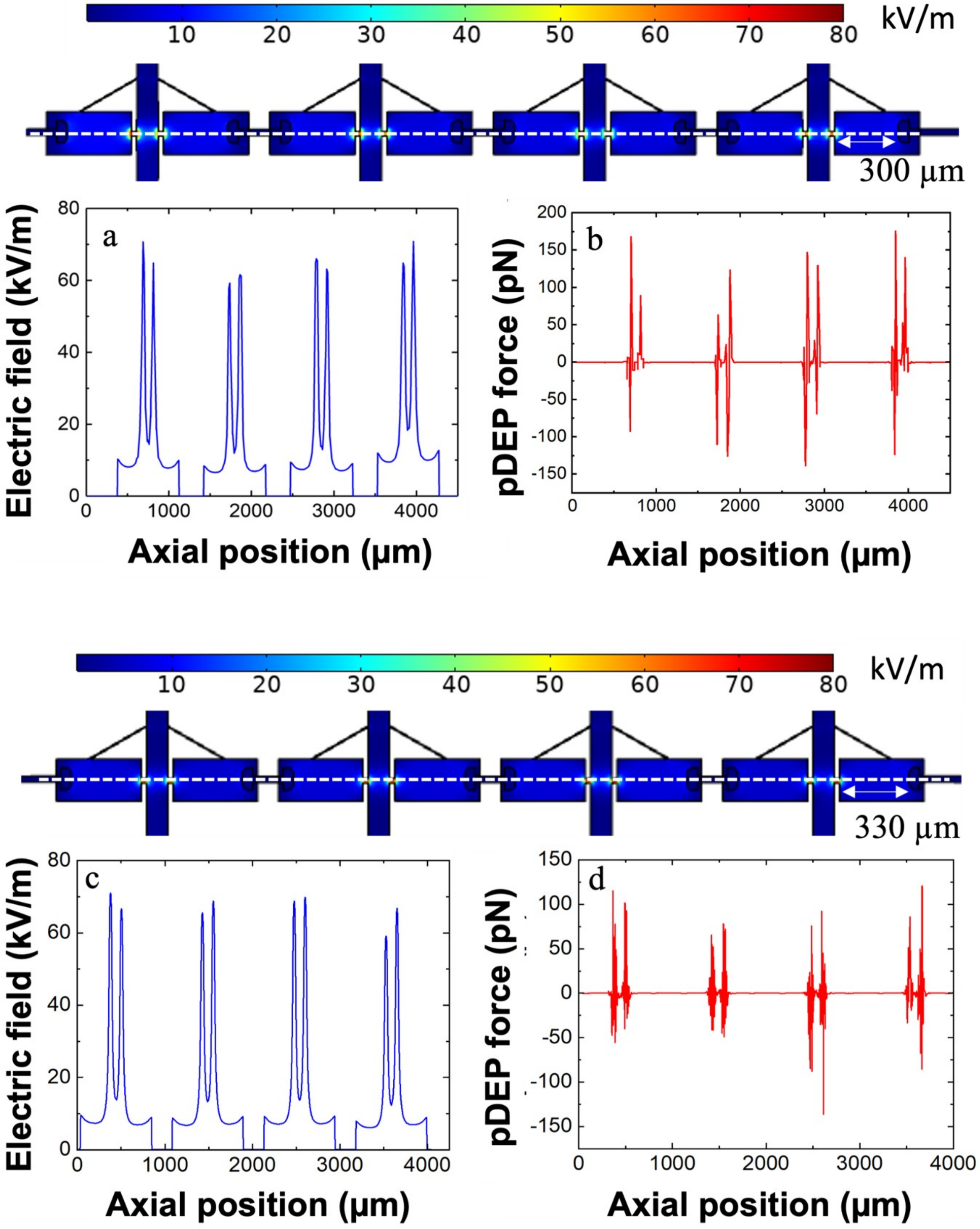

Figure S8. Numerical simulations for (a, c) electric field strength and (b, d) pDEP force along a cutline running through the center of the micropocket and drawn across the entire width of the device for two different pocket-to-electrode gaps, 300 and 330 μm, respectively. (Four-parallel channels).

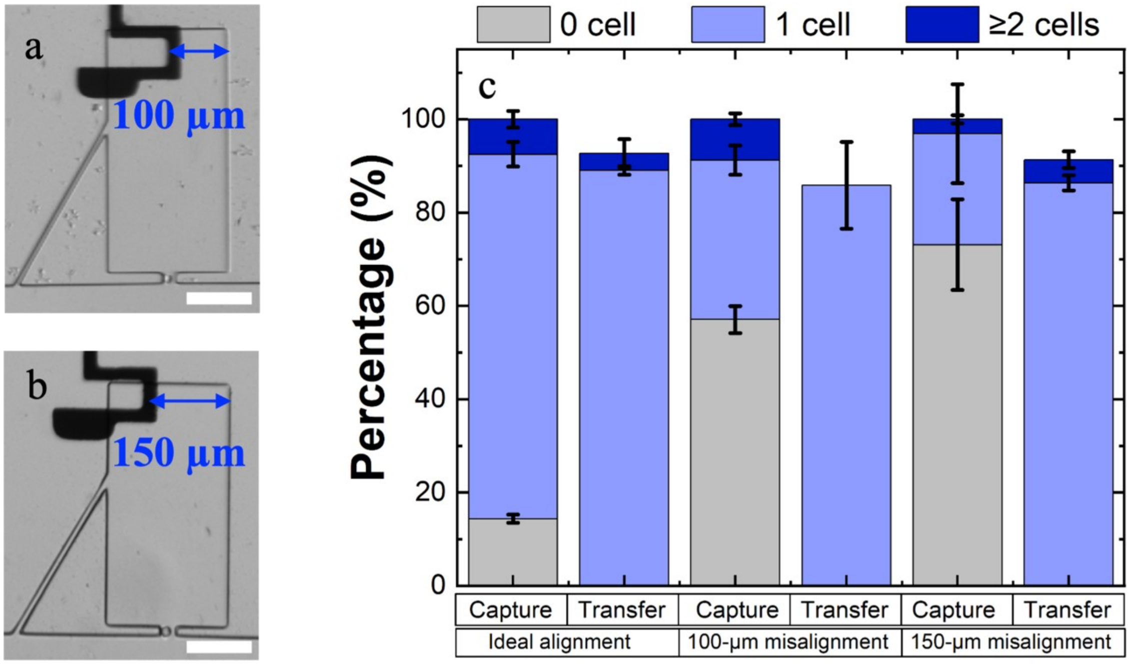

Figure S9. Brightfield micrographs showing cell capture in micropockets with horizontal misalignments of (a) 100 µm (b) 150 µm. (c) The bar graph showing the percentage of empty, singly and multiply occupied pockets and out of the occupied pockets, the percentage of singly and multiply transferred cells as a function of horizontal misalignment as compared to a perfectly aligned paddle electrode presented in Figure 5 of the main text. All electrodes aligned at micropocket-to-electrode gap of 300 µm. Cell concentration 8 $\times 10^5$ cells/mL, applied voltage 66.2 $V_{p\text{-}p}$, flow rate 90 nL/min. Scale bar, 100 μm.
